# Medical education in Bangladesh from Student and Teacher’s Perspective: Impact and challenges of the COVID-19 pandemic

**DOI:** 10.12688/mep.19761.2

**Published:** 2024-02-05

**Authors:** M. Wakilur Rahman, Md Mahfuzul Hasan, Md. Salauddin Palash, Md Asaduzzaman

**Affiliations:** 1 Professor, Department of Rural Sociology, Bangladesh Agricultural University, Mymensingh, Dhaka Division, 2202, Bangladesh; 2 Department of Agribusiness and Marketing, Bangladesh Agricultural University, Mymensingh, Dhaka Division, 2202, Bangladesh; 3 Professor, Department of Agribusiness and Marketing, Bangladesh Agricultural University, Mymensingh, Dhaka Division, 2202, Bangladesh; 4 Assistant Professor, Department of Rural Sociology, Bangladesh Agricultural University, Mymensingh, Dhaka Division, Bangladesh

**Keywords:** Blended System, Covid-19, Online education, SWOT-analysis

## Abstract

**Background:**

In low- and middle-income countries like Bangladesh, where medical education faces a range of challenges-such as lack of infrastructure, well-trained educators, and advanced technologies, abrupt changes in methodologies without adequate preparation are more challenging than in higher-income countries. This was worsened during the COVID-19 pandemic and these challenges have resulted in a change in medical education methodology. This study assesses the medical education procedure, impacts and adaptation strategies and challenges of the COVID-19 pandemic in the medical education system of Bangladesh from learners' as well as educators' perspectives.

**Methods:**

The study collected data from 22 Medical Colleges/Universities across 18 districts of eight divisions using quantitative and qualitative methods. A total of 408 samples were collected consisting of 316 from students and 92 from medical teachers. Descriptive analysis and probit model were performed for obtaining results.

**Results:**

The efficacy of online learning was questionable, but results showed that it was more effective for theory classes (92.4%) followed by clinical classes (75.63%) and the efficacy rate was low for practical classes (54.11%). All types of classes (theory, practical and clinical) are currently using mixed methods to some extent in medical education in Bangladesh. Regarding impacts and adaptation strategy, approximately 75.3% of the students surveyed expressed their acceptance of online education. Over 80% of the participants acknowledged the advantages of online learning, highlighting the freedom to learn from home, cost and time savings, and avoiding physical closeness with other students as major benefits.

**Conclusions:**

To address future challenges like the COVID-19 pandemic in medical education in Bangladesh, a comprehensive policy approach such as strengthening technological infrastructure, promoting blended learning approaches, enhancing faculty training and support, integrating telemedicine into the curriculum, and continuously evaluating and improving policies and interventions can enhance the resilience of its medical education system, and prepare for future challenges.

## Introduction

The severe acute respiratory syndrome coronavirus 2 (SARS-CoV-2) caused the COVID-19 pandemic, which started in late 2019 and spread rapidly over the world and resulted in millions of illnesses and fatalities. The medical education and healthcare systems throughout the world have been severely disrupted by COVID-19 ( Woolliscroft, 2020). As a result of the virus's high contagiousness, it has become difficult to have regular lectures, which has affected the research and patient-based teaching that form the basis of medical school ( Sklar, 2020). The possibility that medical students can come into touch with the virus while receiving their medical training and then disseminate it to the public is one of the issues as well ( Khasawneh *et al.*, 2020). As the world grappled with the challenges posed by the rapid spread of the novel coronavirus, educational institutions faced unprecedented disruptions and had to adapt to new modes of teaching and learning ( Garcia-Morales *et al.*, 2021). The disruption caused by the pandemic has had far-reaching consequences for medical education systems worldwide. For both students and instructors, switching from conventional in-person learning to distance learning platforms presented a number of difficulties ( Selvaraj *et al.*, 2021). The closure of medical colleges and universities in response to the pandemic has led to a disrupted learning environment for medical students. Traditional classroom teaching methods have been replaced by remote learning platforms, posing challenges in terms of adapting to new technologies and maintaining the quality of education ( Maatuk *et al.*, 2022).

Assessment is influenced by both teaching and learning to gauge student and instructor effort ( Kellough & Kellough, 1999). People's well-being and, in fact, their ability to maintain good health in the future are dependent upon the caliber of medical graduates, who in turn are dependent upon the caliber of medical education and evaluation. The overall goal of education may be divided into three categories: knowledge, skills, and attitudes, or what we know, do, and feel. We define knowledge as the entirety of cognitive processes, ranging from simple data recall to comprehension and problem-solving skills. Nevertheless, medical teaching and learning are more complex than other educational programs ( Howley & Wilson, 2004). Apart from learning, they also need to improve their attitudes and skills. Nonetheless, the prevailing assessment methods in most medical institutions primarily emphasize factual knowledge and are largely centered around memorization ( Agusala, 2021). There is minimal emphasis on evaluating practical skills and little consideration for gauging attitudes or behaviors. Due to travel limitations and institution closures because of geographic distance and other lockdown requirements, the COVID-19 scenario has resulted in a dramatic change in medical education technique around the globe ( Ferrel & Ryan, 2020; Rose, 2020). Hence, it becomes more challenging to assess the skills and attitude domain of medical education.

The abrupt changes in methodologies without adequate preparation are more difficult in low- and middle-income countries like Bangladesh, where medical education faces a variety of challenges, such as a lack of infrastructure, well-trained educators, and cutting-edge technologies ( Luna *et al*., 2014). The situation became more intense during the COVID-19 pandemic. The significant challenges to medical education in Bangladesh include disruption of clinical rotations and increased stress and burnout among students. Due to the pandemic's severity, several creative instructional approaches have been developed throughout the world, the bulk of which make use of a range of digital resources ( Papapanou *et al.*, 2022). In fact, it has also created opportunities for innovation and adaptation, such as the adoption of virtual learning platforms and increased training in public health and epidemiology. Previous global studies confirm that medical education lags behind current capabilities regarding online learning and they suggested a hybrid education with at least 40% of online teaching compared to on-site teaching ( Stoehr *et al.*, 2021). Hence, it is crucial to examine the influence of the COVID-19 pandemic on the state of medical education in nations that already face considerable challenges in delivering high-quality medical instruction. This becomes particularly important as the alterations brought about by the pandemic could profoundly affect the future professional opportunities of both existing medical students and trainee doctors. Furthermore, it is crucial to comprehensively examine the innovative methods employed throughout the COVID-19 pandemic, as they could potentially provide valuable insights for educators in the field of medicine in the times ahead. Therefore, this study intended to identify the factors affecting changing medical education processes and identify the impact and challenges in medical education processes during the COVID-19 pandemic on students and teachers.

## Methods

### Study design

This study design is prepared based on available documents and reports provided by Directorate General of Medical Education (DGME) and consultation with the team. A blend of both quantitative and qualitative methods involving in–depth interviews and key informant interviews (KIIs) were employed for collecting relevant data and information. Accordingly, survey instruments were prepared, pre-tested, and then data were collected from different sources. After collection of data, data was edited, coded, categorized, sub-categorized, and analyzed in connection with the settled objectives.

### Selection of the study area

Selection of the study area is an important step for survey research. DGME project intervention was carried out in 08 divisions and 22 districts of Bangladesh. Twenty-four medical college / university hospitals were selected from 22 districts, that means one institution from each district. It can be found in Table 1.

**Table 1.** Distribution of samples across division and medical college/university.

Division	College/University name	Total	Percent	Division total
Dhaka	Bangabandhu Sheikh Mujibur Rahman Medical University	4	1.27	110
	Colonel Malek Medical college	17	5.38	
	Dhaka Medical College	18	5.70	
	Mugda Medical College	18	5.70	
	Shaheed Suhrawardy Medical College	18	5.70	
	Sheikh Sayera Khatun Medical College	17	5.38	
	Sir Salimullah Medical college	18	5.70	
Chattrogram	Abdul Malek Ukil Medical college	15	4.75	51
	Chattogram Medical College	20	6.33	
	Cumilla Medical College	16	5.06	
Rajshahi	Pabna Medical College	16	5.06	36
	Rajshahi Medical University	3	0.95	
	Shaheed Ziaur Rahman Medical College	17	5.38	
Mymensingh	Mymensingh Medical College	14	4.43	41
	Netrokona Medical College	27	8.54	
Rangpur	Rangpur Medical College	14	4.43	28
	M. Abdur Rahim Medical college	14	4.43	
Khulna	Khulna Medical College	14	4.43	14
Barishal	Sher-e-Bangla Medical College	14	4.43	24
Sylhet	Sheikh Hasina Medical college	11	3.48	22
	Sylhet MAG Osmani Medical college	11	3.48	
	**Total**	**316**		**316**

### Study area and sample size determination

It is not worthwhile to conduct a census covering all medical colleges in Bangladesh. In general, there is a greater homogeneity of the medical education across the institutions especially in admission procedure, learning and exam methods, lecturing, etc. Stratified sampling procedure will be adopted for the study. In this study, the sampling frame will be the following:

N= z ^2^ pq/e ^2^ (diff.)

where, N=desired sample, z=1.96, p=0.5, q=1-p, e=margin of error or 0.05 and diff.= design effect or 1.5

So, N = 576 ~ 316 for convenience of allocation for direct beneficiaries. Using the above formula, we get N =316. Therefore, the total sample size for this study is 316 **.** The study collected data from 22 Medical College/University spread to 18 districts of eight divisions.

As well as collecting data from graduate and post-graduate students, 92 teachers were interviewed (adopting KII tool) spread across different medical colleges within different districts and divisions.

### Data collection

Both qualitative and quantitative surveys were performed. This study involved primary surveys, including one-on-one interviews. Respondent category-wise methods are as follows:

S.N associates developed two types of questionnaires: semi structured questionnaire for in–depth interviews and structured questionnaires for key informant interview (KII). These were submitted to the Medical Education and Health Manpower Development (DGME) for reviewing the questionnaire. After review from Medical Education and Health Manpower Development (DGME), S.N Associates incorporated the comments and suggestions of Medical Education and Health Manpower Development (DGME) in the questionnaire and again submitted for approval of the questionnaire from Medical Education and Health Manpower Development (DGME). S.N associates organized pre-tests for comments and suggestions from out of the study area. A total of 15 data enumerators were recruited by SN Associates as data enumerators. All the field staff were recruited among qualified and experienced staff who have participated in at least three studies of a similar nature with S.N associates or another agency. There are a number of data collectors associated with S.N Associates, who worked in different projects conducted by S.N associates. A comprehensive three-day long training workshop on “Data Collection Procedure” was performed in which enumerators spent one day in the field (pre-testing interview schedule). Attempts were made to ensure a uniform pattern in administering the survey. The training plan gave more emphasis on skill training on the real situation rather than classroom training. Data was collected by the enumerators through face-to-face interview under direct supervision of a quality control team. The qualitative information was collected through Key Informant Interview (KII). The KIIs were carried out with medical college/university teachers of under-graduate level and post-graduate level, representative of program manager and service providers of medical college/university hospital. Data collection activities were performed during 15 April, 2023 – 15 May, 2023. The supervisor helped the enumerators/data collectors in locating the facilities, and respondents and ensure quality through supervision. We deployed 5 such teams for the study. The quality control officer and the other team members of the study, who constantly remain in the field, act as facilitators and solve day to day problems that may arise in the field. The QCO physically verified whether the interviewer completed the questionnaire by interviewing the right respondents in the right institutes and respondents by asking the questions.

### Ethical statement

We affirm our commitment to upholding ethical principles throughout the research process. Prior to participation, all student and teacher participants were provided with comprehensive information about the research objectives, procedures, potential risks, and benefits. They were given the opportunity to ask questions and provide voluntary informed consent to participate in the study and also, we ensured them that the accessibility of data would be limited to the research team, and any data stored electronically are protected with appropriate security measures. Ethical approval was gained from Ethical Standard of Research's Committee, Bangladesh Agricultural University and the ethical approval number is 77/BSERT.

### Data processing

Data processing comprises documentation of schedules, editing, coding and computerization, generation of analytical tables, and matching of data. This is well accepted that editing of the collected raw data is a very important task and data quality tremendously improves with efficient editing. We make double entry of 100% data for the sake of ensuring the quality of data entry. Double entry ensures proper cleaning and validation of data by comparing and crosschecking the computerized double output. The first entry matches the second entry for removing inconsistency with internal consistency checks. Moreover, inconsistency check was done between inter-related questions. In addition, tables were generated for all variables for checking range, consistency and quality control of all variables of the data sets.

### Data analysis

After accomplishing the questionnaire survey editing and coding of the collected data were processed at S.N Associates Office. Data analysis was done using SPSS, Stata and Microsoft Excel. Descriptive statistics were used to generate statistical measures such as averages, percentages, ratio, frequency, etc. and Multinomial logit regression model was applied to estimate the influencing factor on medical education particularly theory and practical classes. It is provided in Table 2.

**Table 2.** Methods of data collection.

Information source	Technique/method
Medical college/university student of under-graduate level and post-graduate level.	Quantitative survey: Face to face in-depth interview through structured questionnaire
Medical college/university teacher of under-graduate level and post-graduate level. Program manager and service providers of medical college/ university hospital	Qualitative survey, key informant interview (KII)

## Results and discussion

The COVID-19 epidemic has caused the worst disruptions to educational systems in human history. Everything was locked down across the globe, and nobody ventured outdoors absent of an emergency ( Tadesse & Muluye, 2020). As a result, every type of educational institution shut down, which had a significant negative impact on learners. This study intends to show how the COVID epidemic affected medical education and the future adaptation strategies that are described below.

### Impact of COVID-19 on students’ learning and teachers

**Figure 1.**
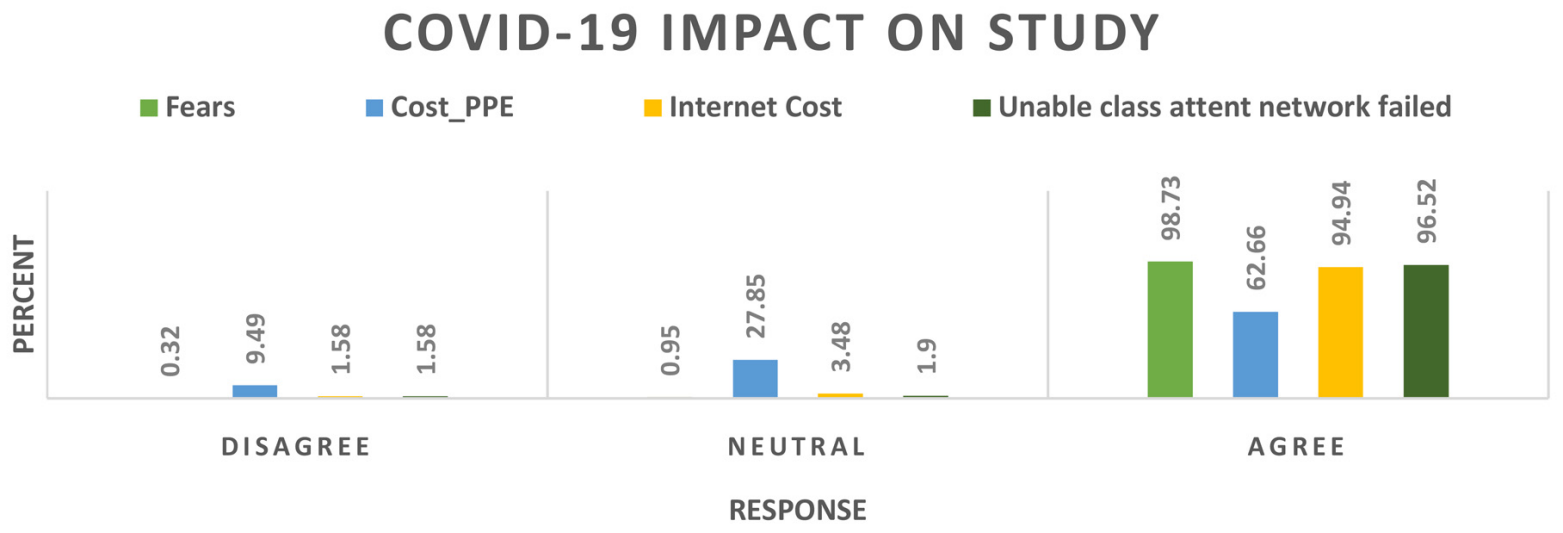
COVID-19 impact on students’ learning.

Medical students' educational experiences have been significantly impacted by the COVID-19 epidemic. One of the most notable changes has been the shift from traditional in-person classes to online learning formats. The hands-on practical experience that is crucial for medical training has been limited due to restrictions on clinical rotations and in-person laboratory sessions. Medical students have had to adapt to virtual simulations and remote learning methods, which may not fully replicate the depth of practical learning they would have gained in traditional settings ( Bashir *et al.*, 2021). This reduced exposure to real-life patient interactions and medical procedures has been a significant adjustment for students. Moreover, it increases the cost of education substantially. For instance, more than 95% of students feel obligated to purchase personal protective equipment (PPE) which increases their expenses. Additionally, if the network goes down, they are unable to attend classes even after making these purchases. Overall, approximately 62.66% of students have concerns related to COVID-19, while 9.49% are not particularly worried about it ( Figure 1).

**Figure 2.**
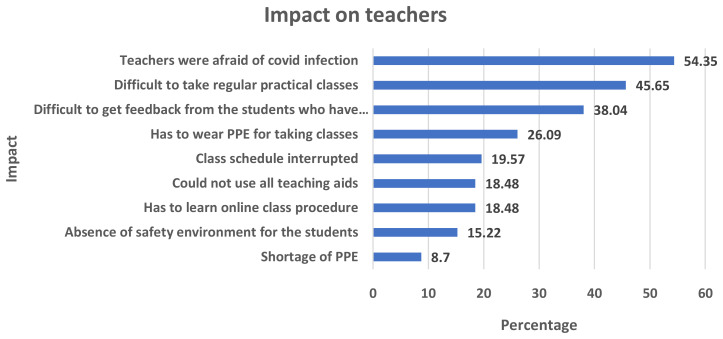
COVID-19 impacts on medical teachers.

The COVID-19 epidemic had a major effect on medical educators. They have had to rapidly adapt to online instruction, requiring them to learn new technologies and adjust their teaching methods (18.48%). Practical and clinical training has been disrupted 45.65%), leading to the exploration of virtual alternatives. Teachers face the difficulties of getting feedback from the student during the COVID period which posited the third rank 38.14%) among all impacts. The pandemic has also taken a toll on the mental health of medical teachers, with increased stress and concerns about personal safety (54.35%). Despite the challenges, the pandemic has spurred innovation in medical education and provided opportunities for professional growth ( Figure 2). Medical teachers have shown resilience and dedication in ensuring the continuity of education during these unprecedented times.

From the viewpoint of the professors, medical students learning system were significantly affected by the COVID-19 epidemic. The sudden shift to remote learning has posed challenges in maintaining the same level of engagement and interaction with students (35.87%). In addition, education cost of the students increased (41.030%) due to maintaining the 4G internet connection cost for online learning methods. It is the topmost impact of COVID-19 on the students of medical education ( Table 3). Teachers have had to quickly adapt their teaching methods to online platforms, navigating technical issues and finding innovative ways to deliver lectures and facilitate discussions. The loss of in-person clinical experiences has affected students' practical training (17.39%), requiring teachers to explore alternative approaches such as virtual patient encounters and case-based discussions. Additionally, teachers have faced the task of providing emotional support to students who may be experiencing increased stress and anxiety during this challenging time (26.09%). Despite the difficulties, teachers have shown resilience and dedication in ensuring the continuity of medical education and supporting their students' learning journey in the face of the pandemic.

**Table 3.** COVID-19 effects on student learning (teachers’ perception).

Impact	Frequency	Percent
Education cost increased due to internet cost	38	41.30
Details discussion was not possible in online class	33	35.87
Many students become depressed	24	26.09
Access to internet was mandatory for class	23	25.00
Students were irregular in class	16	17.39
Practical and clinical class were hampered	16	17.39
Students lost consistency in learning	15	16.30
Students stopped coming to the college considering their safety	12	13.04
Interest on learning hampered	12	13.04
They can't join physically in the class	8	8.70
Has to leave hostel and stay at home	7	7.61
Exam scheduled has changed	1	1.09
**Total respondents**	**92**	**100.00**

### Challenges faced by the students and teachers

Since the COVID-19 pandemic, medical educators have faced several challenges for continuing the teaching and learning activities. Despite the initiatives by education leader and faculty, the mindset was not so ready to adjust with the changing circumstance. It is apparent from Table 4 that, out of 12 identified challenges, a greater percentage (62.70%) of the students reported that ‘fear towards COVID-19’ was so high during COVID-19 pandemic. In fact, this is a usual case as all human beings were afraid to be the victim of the COVID-19 pandemic. On the other hand, a few percentages (15.51%) of the students reported ‘safety measure for the physical classes’ was not that big challenge for them during COVID-19.

**Table 4.** Challenges faced by the students during COVID-19.

Challenges	Response (%)			
	Limited extent	To some extent	High extent	Very high extent
Fear towards COVID-19	0.60	3.50	33.20	62.70
Depression of teachers and students	0.00	8.20	54.50	38.30
Challenges of course continuation	3.80	12.97	63.92	19.30
Maintaining social distance	4.11	21.20	50.63	24.05
PPE supply interruption	6.65	12.03	36.08	45.25
Safety measure for physical class	24.68	41.14	18.67	15.51
Inadequate facilities	9.18	40.19	34.81	15.82
Limited practice physical distance at hostel	8.54	21.20	41.77	28.48
Interruption of clinical practical class	2.22	18.04	44.94	34.81
Limited experience of e-learning	7.28	21.20	52.53	18.99
Course continuation and suspension	2.22	40.51	43.67	13.61
Keep attention in the class	2.22	39.87	31.01	26.90

**Figure 3.**
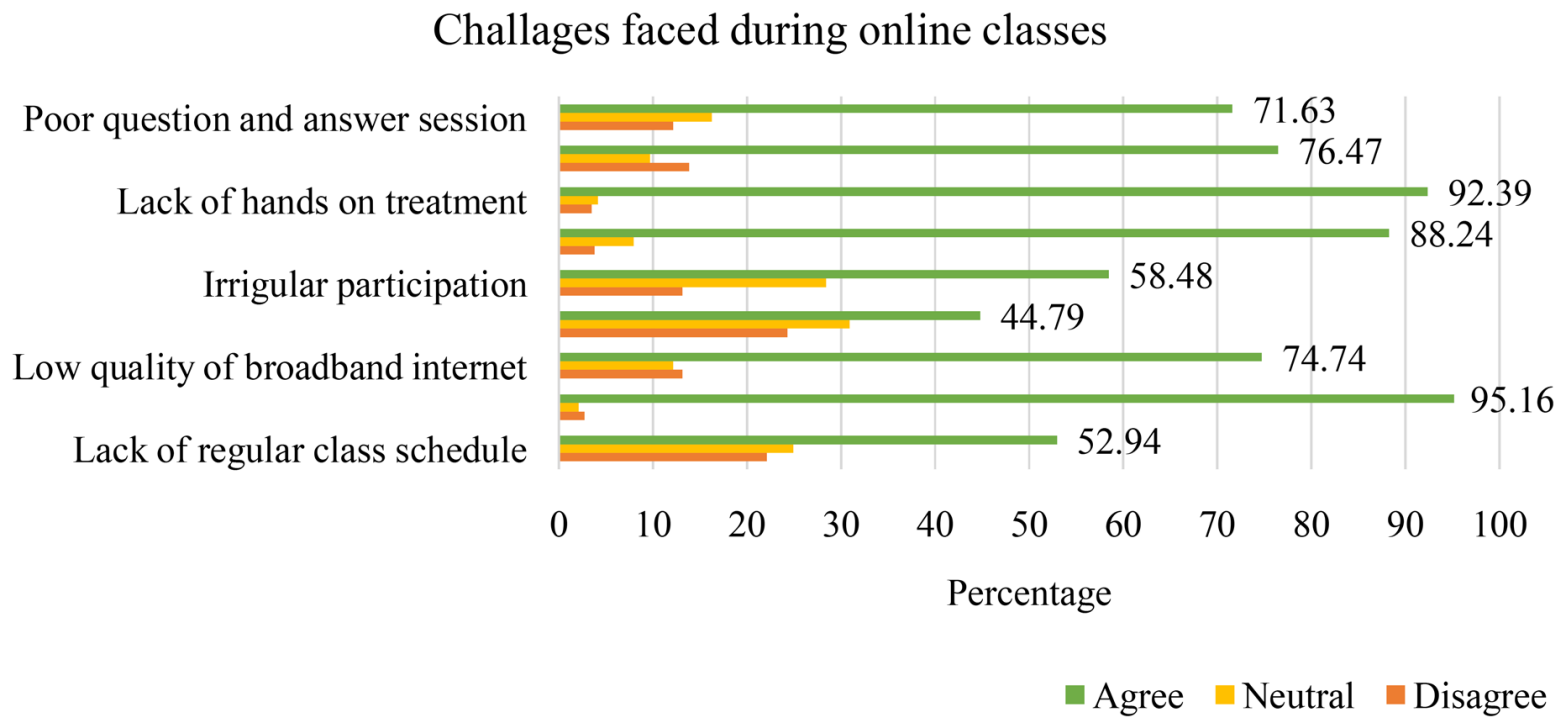
Challenges faced during online classes.

As online class was introduced, many challenges were faced by the students during online classes. One of the pre-requisites of online class is the access to internet. Hence, it is evident that about 91% of respondents faced problems during their online classes. More importantly, it is reported that 95.16% of students complained inadequate internet service in the village was the major problem. Other problems included lack of reliable treatment (92.39%), practical lab invigilation not being possible (88.24%), difficulty drawing teachers’ attention (76.47%), low quality broadband internet (74.74%) etc. ( Figure 3). No doubt the above issues hinder online education. Despite such challenges, students got the opportunity to interact with teachers and got some extent of learning through online class.

Not only the medical students but also the faculty members faced several challenges during the COVID pandemic. The challenges are listed in Table 5 based on the responses of the teachers and ranked accordingly. Students were ‘less interested due to COVID fear’ was ranked first with about 24% of the respondents mentioning this. Other challenges included maintaining social distance, limited orientation about online class, providing instruction to attend online class and following the lecture, requiring online class teaching materials, providing financial assistance to students in need etc. The challenges are many as online education systems have only recently been introduced in medical education. However, within a couple of months the teachers were able to overcome the challenges and conduct the classes in an innovative way as mentioned by many of the KII participants. In fact, there was a co-learning environment created where one faculty member helps others to manage the online classes. 

**Table 5.** Challenges faced by the teachers during the COVID-19 pandemic for medical education.

Challenges	Frequency	Percent	Rank
Students were less interested due to COVID fear	59	23.79	1
Maintain social distance	30	12.10	2
Limited orientation about online classes	23	9.27	3
Provide instruction how to attend on-line class	22	8.87	4
Encourage students to follow lecture	22	8.87	4
Lack of appropriate technology at the beginning	20	8.06	5
Bring students in online class those live in the remote’s location	15	6.05	6
Had to support and encourage those had no smartphone	14	5.65	7
Teacher pay to cost of internet at the beginning	11	4.44	8
Limited online teaching aids	9	3.63	9
Provided financial assistant to the students	8	3.23	10
Mental and technical knowhow of online class	8	3.23	10
Practical class was so difficult	4	1.61	11
Delayed on supplying PPE	2	0.81	12
Proper monitoring was not possible	1	0.40	13

### Steps taken for overcoming the challenges

**Figure 4.**
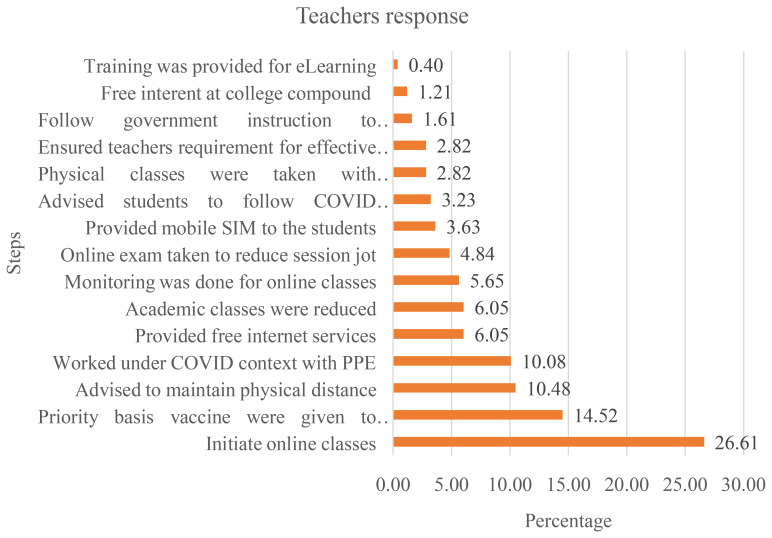
Steps taken to overcome the challenges perceived by teachers.

To continue medical education during the pandemic, medical institutions took several initiatives. Sample teachers have mentioned 15 steps for overcoming the barriers ( Figure 4). Just above 26% of the teacher supports on initiate online class immediately and 14.52, 10.48, 10.08, 6.05, 6.05, and 5.6% teachers also gave priority on vaccine provided, advise to maintain physical distance, work under COVID context maintaining PPE, provide free internet services, academic class reduces, monitoring the online class respectively.

Medical institutions in Bangladesh have responded to the COVID-19 outbreak by putting in place a number of preventative measures to stop the virus's transmission and guarantee the security of the students, teachers, and staff. When surveying medical students on their opinions regarding these initiatives, over 60% of respondents expressed agreement with various statements. These actions encompassed overseeing virtual lectures, enforcing social distancing protocols, supplying personal protective gear like masks, organizing online learning tools, delivering medical care to educators and students impacted by the virus, arranging transportation for staff, enforcing preventive measures to manage virus transmission, documenting hands-on instructional sessions, and administering COVID-19 vaccinations to students.

**Figure 5.**
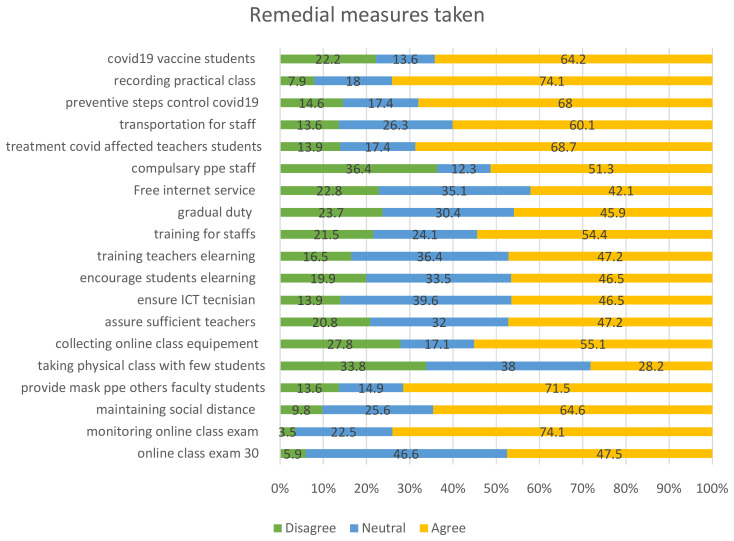
Remedial measures taken by the institutions.

On the other hand, there were certain percentages of respondents who expressed disagreement with these statements. Approximately 3.5% disagreed with monitoring online classes, 9.8% disagreed with maintaining social distance, 13.6% disagreed with providing mask PPE, 27.8% disagreed with collecting online class equipment, 13.9% disagreed with treating Covid-affected teachers and students, 13.6% disagreed with providing transportation for staff, 14.6% disagreed with the preventive steps to control COVID-19, 7.9% disagreed with recording practical classes, and 22.2% disagreed with providing COVID-19 vaccines to students. In contrast, when asked about the possibility of physical education classes with fewer students, only 28% of respondents agreed, while 39% disagreed with this statement which Figure 5 clearly demonstrates this.

### Factors affecting medical education

Multinomial logit regression model was applied to estimate the influencing factor on medical education particularly for theory and practical classes. Only significant variables are presented in Table 6 and Table 7. The calculation shows that, by holding all other variables constant, a one-unit increase in study year for undergraduate students sitting at a distance of three feet would be predicted to result in an increase of 1.014 units in the multinomial log-odds for choosing a three-foot distance over a direct approach. The multinomial log-odds for choosing 3 feet distance to direct would be projected to drop by 2.63 units while maintaining all other factors in the model constant for students who choose to attend class from home, where a one-unit increase in home attendance is equivalent to preferring 3 feet of seating distance to direct class. Again, the quality of electronic device has a positive association with online class preference over physical class. According to the estimation, if the quality of electronic devices for online to direct class increased by one unit, all other model variables would be held constant, and the multinomial log-odds for choosing online to direct class would be predicted to increase by 3.21 units.

**Table 6.** Influential factors in medical education processes changed specifically for theory classes.

**Dependent variable**	Medication education method			
**Reference category**	**Direct education method**			
**Decision variable**	Study year, attending class from home, Additional cost of buying data, Inadequate student participation, Transportation cost saving, Lack of real medical education, Difficulty drawing teacher’s attention, Less mental satisfaction, Quality of electronic devices			
Education method	Significant variable	Coef. Std. Err.	z	P>z
**3-feet distance**	Study year	**1.013**	**2.02**	**0.043**
	Attending class from home	**-2.634**	**-1.65**	**0.099**
**Online**	Quality of electronic device	**3.214**	**2.26**	**0.024**
**Blended**	Study year	**-0.372**	**-2.33**	**0.02**
	Transportation cost saving	**-0.665**	**-1.69**	**0.09**
	Lack of real medical education	**1.033**	**2.00**	**0.046**
Number of obs. = 316	LR Chi ^2^ (27) = 57.50			
Prob > Chi ^2^ = 0.0006	Pseudo R ^2^ = 0.1190			

**Table 7.** Influential factors in medical education processes changed specifically for practical classes.

**Dependent variable**	Medication education method			
**Reference category**	**Direct education method**			
**Decision variable**	Study year, attending class from home, Additional cost of buying data, Inadequate student participation, Transportation cost saving, Lack of real medical education, Difficulty drawing teacher’s attention, less mental satisfaction, Quality of electronic devices, Reduce practical class, Physical visit of lab and ward			
Education method	Significant variable	Coef. Std. Err.	z	P>z
**3-feet distance**	Attending class from home	**-3.463**	**-2.08**	**0.037**
	Physical visit of lab and ward	**-2.689**	**-1.78**	**0.075**
**Online**	Additional cost of buying data	**5.467**	**1.97**	**0.049**
**Blended**	Study year	**-.2602**	**-1.65**	**0.099**
	Transportation cost saving	**-1.274**	**-2.46**	**0.014**
	Lack of real medical education	**1.056**	**1.67**	**0.095**
	Reduce practical class	**-2.047**	**-2.61**	**0.009**
Number of obs = 316	LR Chi ^2^ (33) = 72.21			
Prob > Chi ^2^ = 0.0001	Pseudo R ^2^ = 0.1504			

The multinomial log-odds for choosing blended over direct classes should fall by 0.373 and 0.665 units, respectively, for a blended education system, according to estimation of undergraduate study years and transportation cost saved. This is true even if all other model variables are held constant. However, in respect to lack of reliable treatment, the scenario is the opposite. While maintaining all other model variables constant, it would be predicted that the multinomial log-odds for favoring blended over direct learning would increase by 1.03 units in the absence of a trustworthy treatment by one unit.

Table 7 outlines the factors associated with practical classes that impact changes in the education system. Participating in practical classes remotely and supervising practical labs have been subjected to multinomial logit estimation to assess the effect of a one-unit increase in these variables, assuming that other model variables remain unchanged. The results indicate that a one-unit increase in remote class attendance is linked to an anticipated decrease of 3.46 units in the multinomial log-odds for favoring a 3 feet distance over direct interaction. Similarly, a one-unit increase in lab invigilation imposition corresponds to an expected decrease of 2.69 units in the multinomial log-odds to determine a 3 feet distance over direct interaction, while keeping all other model variables constant.

If a subject has to increase the cost of buying data by one unit, the multinomial log-odds for online to direct learning would be expected to increase by 5.47 units while holding all other variables in the model constant.

In the blended education system, if the transportation cost and practical class reduced by one unit, the multinomial log-odds for a blended to direct education system would be expected to decrease by 0.26, 1.27 and 2.05 units respectively while holding all other variables in the model constant. On the other hand, for lack of reliable treatment, if it increased one unit for lack of reliable treatment, the multinomial log-odds for blended to direct learning would be expected to increase by 1.06 units while holding all other variables in the model constant.

### Future initiatives

**Figure 6.**
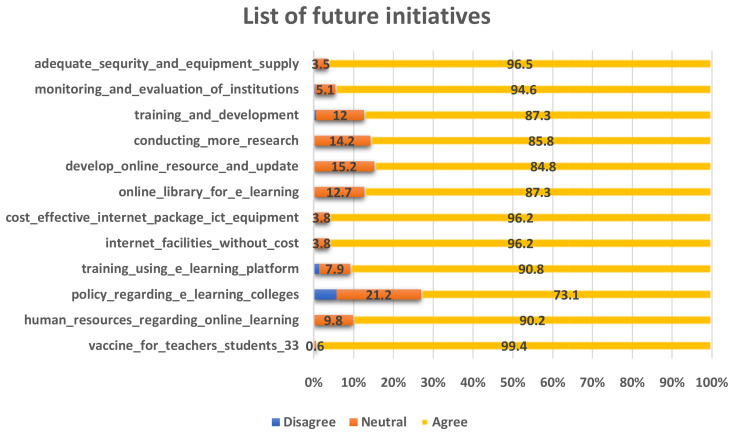
List of future initiatives that need to be taken by the institutions to face the pandemic.

To effectively address future challenges like the COVID-19 pandemic, medical institutions need to adopt and implement various future initiatives. This is depicted in Figure 6. These initiatives should prioritize the safety and well-being of students, faculty, staff, and the wider community while ensuring the continuity of education and healthcare services.

When inquiring about their views on addressing future challenges, the majority of respondents expressed a positive outlook in response to all the queries. The only exception pertained to the policy regarding the e-learning system in colleges. In this regard, 5.7% of respondents disagreed, while 21.2% remained neutral in their stance.

## Conclusion

Bangladesh pupils and instructors have experienced severe effects from the COVID-19 epidemic, prompting quick adaptation measures. The abrupt switch to online learning has caused interruptions for students, who now confront difficulties with access to technological advances, internet access, and a change in the learning environment. Teachers have had to quickly adapt their teaching methods to remote platforms, finding innovative ways to engage students and ensure the continuity of education. In spite of these obstacles, both students and instructors have shown resiliency, adaptation, and a strong dedication to learning. They have embraced digital tools and resources, explored virtual platforms for interactive learning, and collaborated to overcome obstacles together. As a result of the pandemic, technology use in education has increased, giving rise to the creation of new ways and capabilities. Moving forward, the lessons learned from this experience can inform future strategies to strengthen the education system in Bangladesh, ensuring it is better equipped to handle similar crises. It is crucial to continue investing in infrastructure, digital literacy, and professional development opportunities for both students and teachers. By doing so, medical institutions can build a more resilient and inclusive education system that prepares students for the challenges of the future.

A thorough policy strategy is required to handle upcoming issues like the COVID-19 epidemic in Bangladeshi medical training. This includes developing an emergency preparedness plan, strengthening technological infrastructure, promoting blended learning approaches, enhancing faculty training and support, integrating telemedicine into the curriculum, implementing effective assessment strategies, establishing collaboration and information sharing among institutions, prioritizing student well-being and mental health support, and continuously evaluating and improving policies and interventions. By adopting these measures, Bangladesh can enhance the resilience of its medical education system, ensure uninterrupted learning during crises, and prepare for future challenges.

### Policy implications

The COVID-19 pandemic issue has opened up a new opportunity for adopting an appropriate educational practice that includes teachers, students, and affiliated health professionals and values professional skills and ethics from a humanistic ethical perspective. A direct, hands-on clinical experience should continue with the conventional ways to develop their abilities and expertise as part of the quality of medical education in order to install such graduate traits. To counteract and resolve the crisis brought on by the COVID-19 pandemic, medical schools and other health science institutions must adapt their curricula and educational strategies using new remote learning modalities, such as extended reality technology, e-learning tools, and simulation facilities.

Additionally, medical institutions should implement best practices for an active transition to online learning and evaluation based on the lessons acquired from this pandemic knowledge. It is also advised to maintain blended learning in the post-COVID-19 era with a sizable portion of online modes since conventional and online virtual learning combinations are thought to be extremely stable and long-lasting. Additionally, medical educators should develop the proper policies or safety precautions to undertake educational activities without endangering the security and well-being, of medical education.

## Data Availability

Figshare: Medical education in Bangladesh from student and teacher’s perspective: Impact and challenges of the COVID-19 pandemic https://doi.org/10.6084/m9.figshare.24114390 ( Hasan, 2023) This project contains the following underlying data: Data entry both Under _ post graduate.xlsx (this file contains different the raw quantitative data for variables) Table.docx (this file contains all the table which is used for this research paper) Please note that the responses to the qualitative survey questionnaire are in Bangla. Specific quotes or themes can be translated in the xlsx data file. Figshare: Medical education in Bangladesh from student and teacher’s perspective: Impact and challenges of the COVID-19 pandemic https://doi.org/10.6084/m9.figshare.24114390 ( Hasan, 2023) This project contains the following extended data: KII_guideline_Teachers.docx (This file contains the Introduction and Consent as well as questionnaire) Questionnarie_Final Draft_Students.docx (In-depth Interview (IDI) Questionnaire) Questionaire Medical Education KII.docx Figure.docx Data are available under the terms of the Creative Commons Attribution 4.0 International license (CC-BY 4.0).
