# Supplementary material for: Medical education in Bangladesh from Student and Teacher’s Perspective: Impact and challenges of the COVID-19 pandemic
Source: MedEdPublish (2016). 2024 Feb 5;13:209. Originally published 2023 Oct 5. [Version 2] doi: 10.12688/mep.19761.2 (PMC11058450; doi:10.12688/mep.19761.2)
Supplement: Peer review history

# Reviewer report: Reviewer response for version 2

**DOI:** 10.21956/mep.21602.r36365

**Published:** 2024-04-29

**Authors:** Syeda Sadia Fatima

**Affiliations:** 1 Aga Khan University, Karachi, Pakistan

Reviewer Comment:

 The authors have presented a comprehensive assessment of the impact of the COVID-19 pandemic on medical education in Bangladesh, examining both learner and educator perspectives. The paper is commendable for its clear methodology and presentation of results. However, I suggest the authors consider incorporating the following to improve the paper.

 Minor:

 A graphical summary representing the study design, methods and key results with areas of future work can be added to the paper. 

 Major:

 A limitation section of the study. This would provide important context for interpreting the findings and acknowledge potential areas for further research or improvement.

 I recommend the inclusion of examples from other SAAP countries in the discussion section. This would allow for a broader comparison of the challenges and adaptation strategies faced by medical education systems across the region. By doing so, the authors could offer valuable insights into common issues and potential collaborative solutions among LMIC universities.

 Suggest a few solutions based on existing medical education theories and contextualize them to fit the need of the area.

 While the paper is well-written and informative, addressing these suggestions would enhance its relevance and contribution to the field of medical education in the context of the COVID-19 pandemic.

Have any limitations of the research been acknowledged?

Partly

Is the work clearly and accurately presented and does it cite the current literature?

Yes

If applicable, is the statistical analysis and its interpretation appropriate?

Yes

Are all the source data underlying the results available to ensure full reproducibility?

Yes

Is the study design appropriate and is the work technically sound?

Yes

Are the conclusions drawn adequately supported by the results?

Yes

Are sufficient details of methods and analysis provided to allow replication by others?

Yes

Reviewer Expertise:

Technology-enhanced learning, faculty development, curriculum development, assessment.

I confirm that I have read this submission and believe that I have an appropriate level of expertise to confirm that it is of an acceptable scientific standard.

## References

[1] : Online assessment in undergraduate medical education: Challenges and solutions from a LMIC university. *Pak J Med Sci* .2021;37(4) : 10.12669/pjms.37.4.3948 945-951 10.12669/pjms.37.4.3948 34290764 PMC8281159

# Reviewer report: Reviewer response for version 2

**DOI:** 10.21956/mep.21602.r35977

**Published:** 2024-03-01

**Authors:** Michael Paget

**Affiliations:** 1 University of Calgary, Calgary, Alberta, Canada

Thanks for completing the revisions, no further comments.

Have any limitations of the research been acknowledged?

No

Is the work clearly and accurately presented and does it cite the current literature?

Yes

If applicable, is the statistical analysis and its interpretation appropriate?

Yes

Are all the source data underlying the results available to ensure full reproducibility?

Partly

Is the study design appropriate and is the work technically sound?

Yes

Are the conclusions drawn adequately supported by the results?

Partly

Are sufficient details of methods and analysis provided to allow replication by others?

Yes

Reviewer Expertise:

Undergraduate Medical Education, Academic Technologies.

I confirm that I have read this submission and believe that I have an appropriate level of expertise to confirm that it is of an acceptable scientific standard.

# Reviewer report: Reviewer response for version 1

**DOI:** 10.21956/mep.21175.r35115

**Published:** 2023-11-27

**Authors:** Michael Paget

**Affiliations:** 1 University of Calgary, Calgary, Alberta, Canada

To the research team, I applaud your efforts in trying to understand the challenges for medical education during a difficult time, and including both teachers and students in your research. 

 Some specific feedback:

 “Nonetheless, the prevailing assessment methods in most medical institutions primarily emphasize factual knowledge and are largely centered around memorization.” Perhaps a reference needed for this statement?  

 Some of the limitations of the survey could be addressed by establishing the saturation at each site. We have the number of completed surveys, but what percentage does that represent of each schools students?  

 Spelling mistake in Figure 2 – Teachers were “affaird” of COVID infection

 This doesn’t appear to have data associated with it: “From the viewpoint of the professors, the COVID-19 epidemic has significantly affected the knowledge of medical students.”  

 Table 3: “Students’ loss consistency in learning” may be better stated as “Students lost consistency in learning”.

 For clarity it may be worth re-ordering Figure 5 by rate of disagreement, and re-order the paragraph in ascending order.

Have any limitations of the research been acknowledged?

No

Is the work clearly and accurately presented and does it cite the current literature?

Yes

If applicable, is the statistical analysis and its interpretation appropriate?

Yes

Are all the source data underlying the results available to ensure full reproducibility?

Partly

Is the study design appropriate and is the work technically sound?

Yes

Are the conclusions drawn adequately supported by the results?

Partly

Are sufficient details of methods and analysis provided to allow replication by others?

Yes

Reviewer Expertise:

Undergraduate Medical Education, Academic Technologies.

I confirm that I have read this submission and believe that I have an appropriate level of expertise to confirm that it is of an acceptable scientific standard, however I have significant reservations, as outlined above.
